# Some Types in Gynecological Practice1Read before the Medical Society of the County of Steuben, at Hornell, October 12, 1909.

**Published:** 1910-02

**Authors:** James E. King

**Affiliations:** Buffalo, N. Y.; Adjunct Professor of Obstetrics, University of Buffalo; Attending Gynecologist at the Buffalo General and Erie County Hospitals; Fellow Royal Society Medicine, London, Eng.


### Some Types in Gynecological Practice[1]

JAMES E. KING, M.D., Buffalo, N. Y.

Adjunct Professor of Obstetrics, University of Buffalo; Attending Gynecologist at the Buffalo General and Erie County Hospitals; Fellow Royal Society Medicine, London, Eng.

THE surgical side in the treatment of all pelvic conditions has grown to such an extent, that it leaves but few diseases of the pelvic organs that have not at least surgical possibilities. The brilliant results achieved in pelvic and abdominal surgery

---

1. Read before the Medical Society of the County of Steuben, at Hornell, October 12, 1909.

justify, to a large extent, the confidence placed in it by profession and laity as a means of cure. It can not be said, however, that every result of even the best surgery is all that could be wished. There is a vast difference between an anatomic and a symptomatic cure. The surgeon may feel that his work was well done and that he has accomplished anatomically all that could be desired. The patient, perhaps, may take an entirely different view, justified by a persistence of her old or by acquiring new symptoms. The writer has been interested in such cases for the reason that he has a proportion of such patients of his own to study, as well as cases that have been operated upon by others. Every physician who does surgery or has much surgery done is indeed fortunate if he has not some such patient under his care. It is not the purpose of this communication to take up the question as to why a certain proportion of pelvic or abdominal operations fail to cure, but only to bring to your attention three types of pelvic disease, in which other factors must be taken into account when considering the prognosis and treatment.

It is a well recognised fact that the same pelvic lesion in different women may produce widely different effects. This explains why symptoms, in and of themselves, are of such little value in determining the pathology of the pelvic organs in a given case. Why such variation occurs can not always be explained satisfactorily. In search for an explanation we are often compelled to turn to that rather vague element, entering into the personality of every individual, which is expressed by the term "temperament." What factor or factors are responsible for the different temperaments is an unknown quantity. Whether temperament is dependent entirely upon the individual's mental state, whether it is a problem for physiological chemistry or whether it is possibly dependent upon minute histological variations of the central nervous system, which are not as yet possible of demonstration, are questions which wait to be solved.

There is probably no branch of medicine where more variety may be seen in temperament than in a practice devoted to the diseases of women. Women possessed of a certain impressionable and susceptible temperament will develop, especially with certain pelvic disorders, that peculiar, little understood syndrome which we term neurasthenia. When the writer meets those particular pelvic disorders, the neurasthenic element is sought for and taken into account in the prognosis and treatment of the patient. It is not to be understood that the conditions to be mentioned are the only ones with which neurasthenia may be associated, for indeed there is no condition, pelvic or otherwise, that may not produce it, but in the types to be mentioned, the neurasthenic element will be found in practically every case and it constitutes a very important feature. In a general way, the writer has not found that there is any relation between the character and severity of the pelvic disease, and the symptoms to be expected in the neurasthenic group. The simplest pelvic pathology may be accompanied by the most pronounced neurasthenia, and on the contrary, the most extensive pelvic disease may be associated with no neurasthenic phenomena.

The first type to which attention is asked, is the simple uncomplicated retroversion, a displacement with neither adhesions nor adnexal complications. With such a retroversion the symptoms may be out of all proportion to the actual condition in the pelvis. Every such case should be most carefully scrutinised. The neurasthenic element should be estimated. If this group of symptoms predominate a careful examination should be made to determine the existence of general ptosis of the abdominal contents. It is well to bear in mind that, like the displaced kidney, the displaced uterus is often only an expression and a part of an intestinal ptosis. If such a state of affairs exists, operation for the purpose of correcting the position of the uterus will not give the desired result. It is a question in this class of patients if operation is not only of no benefit, but even indeed harmful. The hospital life and operative procedure intensify the neurasthenic element in these women. On the other hand, if careful search fails to reveal any other cause for the neurasthenia, operation on the uterus will give the most gratifying result. Sometimes in a patient presenting vague, ill defined symptoms, in the course of a general examination the pelvis reveals an unsuspected simple retroversion. This is often at once made the scapegoat. Operation here should not be done until every other possibility has been eliminated. The writer sometimes in such cases replaces the uterus and fits a pessary, which is worn several weeks as a therapeutic test. If the symptoms are relieved operation is advised.

The next type is chronic adnexal disease, with or without retroversion. Here we have a very different state of affairs than that mentioned above. The pathology of all the cases under this type is practically the same with perhaps minor modifications. The tubes are found to be in a state of chronic inflammation. They are thickened, contain no pus and are bound down firmly by old adhesions. The ovaries are often cystic and are more or less involved with the tubes in adhesions. If the uterus be retroverted it too is bound down. This type constitutes a large number of the pelvic invalids, whose ups and downs take them from doctor to doctor in search of relief. The clinical history will usually be found to extend over a number of years with the exacerbations so commonly seen in this class of patients. Many of such women have passed through the "Favorite Prescription" and Lydia Pinkham stage and some through Viava and kindred "cures." When she finally presents herself to the surgeon she is a nervous wreck, disappointed, discouraged and skeptical; a state of mind which is, we must admit, somewhat justified. These patients develop most marked neurasthenic phenomena. In many of the patients of this type the local symptoms may have subsided leaving the neurasthenia to dominate the case. Without entering here into a discussion of the possible influences that produce the neurasthenia, the writer believes that the first step toward a cure is, in a majority of cases, to clean out the diseased adnexa. Good judgment is necessary in determining what may be safely left. As a rule, these are not the cases for conservative surgery. An attempt to leave an unhealthy ovary, either entire or in part, may mean a failure in the symptomatic cure. In the prognosis of such a case after operation, the neurasthenic element plays a very important role. It is desirable that a frank statement should be made to the patient, explaining her pelvic condition and the impress that it has made upon her nervous system. It should be pointed out that surgery can but remove the cause, and that time and treatment together must correct the effect of her years of suffering. With such an explanation, the woman does not expect to convalesce from her operation rid of all her troubles, and she will give much better aid in the further steps necessary for her cure. The keen disappointment experienced by a woman two or three months after an operation, when she realises that she is not well, has its pernicious influence and makes further progress to health more difficult.

Occasionally, in some cases of this type, surgery seems to fail completely. I do not know how one is going to determine whether, after appropriate operation, the neurasthenic element will improve. A case in point was a patient that the writer operated upon and removed both tubes which were thickened and bound down by dense and old adhesions. The indications for operation were pelvic pain and general neurasthenic manifestations. Operation was not followed by improvement. Indeed some of the symptoms were made worse. A little over a year later, in another city, both ovaries were removed. She returned no better. The writer refused to do anything further, but a year or so later some one operated again and about all he left her in the pelvis was her rectum. When last heard of she was in a sanitarium. She may be better now but it is doubtful.

The third and last type for your consideration is endometritis and the conditions so often associated with it. The pathology of this type is usually simple. The cases in mind are such as frequently follow abortion and labor. The so-called "erosions" and laceration of the cervix may be present. Bimanual examination will disclose a tender uterus and even pressure against the body of the uterus through the vagina with the examining finger will elicit tenderness. Sometimes with endometritis alone and often with erosions and lacerations of the cervix, the uterosacral ligaments will be tender and sore. This feature no doubt contributes no small part in the production of the symptoms. Patients of this type present fairly constant pelvic symptoms. There is discharge of course, but the striking feature is the soreness across the lower abdomen. This soreness is intensified by walking or riding, and even standing adds much to the discomfort. With such a pathologic and clinical picture as this, if the condition has existed for any length of time, there will be present the neurasthenic syndrome in varying degrees.

In this connection a very common manifestation is irritability of temper. Such history may not be obtained from the patient, but members of her family will be very ready to testify to it. There is probably no class of gynecological patients that receives more office treatment than this type, and often well directed efforts will effect a cure. When such treatment fails, as it sometimes does, the curet is usually brought into requisition. It is a mistake to assure such a patient that this procedure will end her troubles at once. It may not. The gynecologist sees cases that have been curetted once and sometimes twice without benefit. Here more general treatment will most often give the best results. The writer is skeptical about small lacerations needing repair and, as a rule, would advise against it in these cases. The more extensive tears should, of course, be repaired. The attention should not be so closely directed to the uterus that the woman is neglected.

Of the other many pelvic conditions seen, there are none which so uniformly are associated with neurasthenia. With pus tubes, tumors and abscesses in estimating the prognosis, we have only to obtain an anatomic cure and we are practically assured of a symptomatic one. To speculate as to why these certain pelvic types are so frequently complicated by the neurasthenic syndrome, leads us into a labyrinth of theory, interesting but failing to satisfy. In a general way any prolonged irritation, whether it be mental or physical, in certain individuals will produce it. In many cases it is no doubt a psychological problem. In the majority of cases, it can be attributed to some physical cause; an irritation, perhaps simple in itself, but its prolonged influence finally leaves its imprint on the woman's nervous system. A minor physical ailment is then magnified into a serious condition. When the situation has proceeded thus far the very large mental element in the case must be recognised. It is then that we long for a practical and rational system of mind therapy. The medical profession is beginning to realise how greatly to blame it is, for the existence of the many cults which have for their foundation "mind cure." The writer is confident that one day the medical profession will evolve a psychic therapy, which will aid us in the treatment and cure of our "pelvic neurasthenics."

In conclusion, the writer would bespeak greater attention to the general effect of the pelvic states herein described, to the end that the treatment of these conditions may be along the broad lines necessary to obtain a symptomatic as well as an anatomic cure.

1248 Main Street.

